# Mortality Rates in Femoral Neck Fractures Treated With Arthroplasty in South Africa

**DOI:** 10.1177/21514593221117309

**Published:** 2022-08-03

**Authors:** Jacobus D. Jordaan, Marilize C Burger, Shafique Jakoet, Muhammad Ahmed Manjra, Johan Charilaou

**Affiliations:** 1 Division of Orthopaedic Surgery, Department of Surgical Sciences, Faculty of Medicine and Health Sciences, 121470 Stellenbosch University, Cape Town, South Africa

**Keywords:** mortality rates, neck of femur fractures, hip arthroplasty, South Africa, cementing

## Abstract

**Objectives:**

To investigate the mortality rate for neck of femur fractures treated with arthroplasty at a tertiary level unit in South Africa and to evaluate the effect of known risk factors for mortality in neck of femur fractures treated with arthroplasty in the South African context.

**Design:**

Retrospective cohort study. The main outcome was to determine mortality rates during in hospital stay, at 3 months, 6 months 1 year post surgery. The secondary outcome was to determine factors influencing mortality at 30 days, 6 months and 12 months post-surgery.

**Results:**

Mortality rate was 3.3% in hospital, 5.6% at 30 days and 26.7% at 1 year. Age >79, ASA score >3, and cementing of the femur had statistically increased mortality risk (*P* < .001). Average length of hospital stay was 12.3 ± 5.1 days (range 3.0-41.0 days) with 73% of patients discharged back to pre-hospital home.

**Conclusion:**

Mortality rates after femur neck fracture arthroplasty in South Africa are slightly higher at 1 year compared to international data. However, the rates are comparably low during hospital stay, 30 day and at 6 months post-surgical intervals.

## Introduction

The management of femoral neck fractures (FNF) continues to evoke intense debate and controversy. It has been labelled as “the unsolved fracture” in orthopaedic surgery owing to the high rate of complications and mortality risk, creating a significant socio-economic burden.^1,^^2^

Health care advances have led to an increased global life expectancy resulting in a rising number of fragility fractures. Ratti et al (2013) reported an estimated 29.8% increase in fragility FNF in the United States of America (USA) and Europe between 2000 and 2009.^3^ However, whilst an abundance of literature is available on the management of FNF in high socio-economic countries,^4-6^ there is a significant paucity of literature from middle to low-income countries. In a recent systematic review that compared international mortality trends following hip fractures, the authors noted they could find no published data from Africa during the review period.^7^

Mortality rates associated with FNF are traditionally thought to vary from 10% at 30 days to ∼30% at 1-year post-operatively.^8^ These rates have been reaffirmed more recently by various studies including a review of 40 year mortality rates in the United Kingdom (UK) by Haleem et al,^9^ a review of 31 year mortality rates in Canada by Mundi et al,^10^ and a review of 17 year mortality rates in Spain, with 1-year mortality ranging from 25 to 40% by Guzon-Illescas et al.^6^ Morbidity rates after hip fractures have also remained constant over the last 25 years in Sweden, despite health care advances, as published by Turesson et al.^11^

The timing of surgery, following a hip fracture, is known to have an association with mortality risk.^5,25^ The general consensus and gold standard is that surgery should be performed within 48 hours of hospital admission.^5,6,12,13^ However, delays between injury and admission are not factored into these conventional timeframes. In low to middle-income countries, these delays can be considerable, yet the impact has not been investigated or reported on.

Recently, the first publication to provide specific incidence rates based on ethnicity and gender, within South Africa, reported that hip fractures occur in 68.6 patients per 100 000 population.^14^ South Africa is considered a low to middle-income country and health care is hampered by the quadruple burden of disease of tuberculosis and Human Immunodeficiency Virus (HIV); trauma; high infant and maternal mortality; together with the emerging burden of non-communicable diseases.^15^ In addition, there are large inequalities in terms of access to orthopaedic care in South Africa for a large part of its population.^16-18^

The main purpose of this study was to investigate mortality rates in FNF treated with arthroplasty in a single academic public hospital in South Africa. The secondary aim was to describe and evaluate possible contributing risk factors unique to our population and health care system.

## Methods

A retrospective cohort investigation, including all consecutive patients presenting with a FNF requiring hip arthroplasty between 1 January 2015 and 31 December 2017 to a tertiary hospital in South Africa was performed. Exclusions were pathological fractures, periprosthetic fractures, patients not fit for surgery and patients with incomplete information that could not be validated.

Patient demographics and information related to the mechanism of injury (MOI), surgery and discharge were collected. Mortality data was recorded as date of death according to hospital records and was confirmed and validated by the Department of Home Affairs (DHA) using a national identification number review. Out-of-hospital mortality rates were also retrieved from the DHA and incorporated into the results.

Surgical approach varied between surgeons as part of a teaching platform, between antero-lateral, posterior and direct anterior. Decision making between hemi-arthroplasty (HA) and total hip arthroplasty (THA) was made according to the National Institute for Health and Clinical Excellence (NICE) guidelines: Patients that were independent community mobilisers with normal cognitive function received THA; home ambulators and demented patients received HA; low function non-walkers received a cemented Thompson’s prosthesis.

Choice of femoral cementation was determined by pre-operative radiographic analysis together with intra-operative bone quality assessment. All acetabular components were uncemented.

The patient’s peri-operative risk category was recorded using the American Society of Anaesthesiologists’ classification (ASA) score. The Elixhauser co morbidity index was used to categorize comorbidities of individual patients. Total length of hospital stay (LOS) was calculated from admission into hospital to discharge home, step-down facility or secondary referral base hospital. All-cause mortality was captured as in-hospital, 30 days, 3 months, 6 months and 1 year post-surgery, with mortality being assessed cumulatively during the different time intervals. Mortality confirmation was performed during April 2020 at a mean of 3.6 years post-surgery. Associations between risk factors and mortality were considered at 30 days, 6 months and 12 months post-surgery.

Data was analysed using STATISTICA version 13.0 (StatSoft Inc, Tulsa, OK, USA). Captured clinical and demographic data is presented as mean ± standard deviation or median and interquartile range (IQR). Categorical data is presented as frequencies and counts. Independent *t*-tests or Mann Whitney *U*-tests were used to detect differences between groups of parametric and non-parametric data respectively whilst the Pearson’s chi-squared or Fisher’s Exact test was used to detect differences between categorical variables. Multivariable logistic regression analysis was performed at the 6-month and 1-year timepoints, where the sample size of the number of events (ie, mortality) was large enough. The sample size was determined using the rule of thumb of 10 events per predictor variable considered: as 6 independent variables were investigated, a minimum number of 60 events was required for the analysis. Predictor variables were individually be tested against the outcome of mortality at the given timepoint (ie, 6 months or 1 year) using as previously described. A *P*-value <.2 was be used to select variables to take forward into a multivariable model, and Odds ratios (OR) and 95% confidence intervals are reported. Backward and forward stepwise selection methods was be used to determine a final logistic regression model which includes only predictors with *P* values < .05. Adjusted OR’s and 95% confidence intervals are reported for predictor variables that remained in the final multivariable models.

## Results

Over the 3 year study period, a total of 312 hip arthroplasties were performed of which 9 patients with known pathological fractures and 4 patients that were deemed not fit for surgery and treated conservatively were excluded. Bilateral hip fractures were observed in 2 patients and are reported as separate events. The final cohort comprised 303 joint replacements, of which 69.3% (n = 210) were performed in women and 30.7% (n = 93) in men with a total mean age of 73.8 ± 12.4 years (95% CI: 72.4-75.2; range 37-105 years). Men were significantly younger (68.9 ± 11.7 years) than women (75.9 ± 12.1 years) at presentation (*P* < .001) and left sided fractures were more prevalent, in 57.8% (n = 175) of patients (Table 1).

**Table 1.** Patient Demographic Information.

Demographic variable	All patients, N = 303
Age	73.8 ± 12.4
Gender % female	69.3 (210)
Affected side. % left	57.8 (175)
MOI	
Fall-same level	91.7 (278)
Fall from height	2.0 (6)
Assault	2.0 (6)
PVA	2.0 (6)
MVA	.7 (2)
Unknown	1.7 (5)
ASA score*	
1	5.9 (18)
2	46.5 (141)
3	41.3 (125)
4	5.3 (16)
Unknown	1 (3)
Comorbid conditions	
Hypertension	34.6 (105)
Diabetes	17.8 (54)
Dementia	9.6 (29)
Chronic pulmonary disease	5.9 (18)
HIV	3.0 (9)
Elixhauser comorbidity index score	
<0	4.0 (12)
0-4	65.7 (199)
4-10	24.4 (74)
11-15	3.3 (10)
>15	2.6 (8)

Data is presented as mean ± standard deviation, or as a frequency with count indicated in parentheses. PVA, pedestrian vehicle accident; MVA, motor vehicle accident; ASA, American society of anaesthesiologist classification; MOI, mechanism of injury. *The ASA score of three participants was unknown.

The most common mechanism of injury (MOI) was a low energy fall in 93.7% (n = 284) and patients presented predominantly with an ASA score of 2 (47.0%, n = 141) or 3 (41.7%, n = 125) while most patients had an Elixhauser score between 0 and 10 (Table 1). Surgical approach varied between posterior 61.7% (n = 187), direct anterior 28.4% (n = 86) and antero-lateral 9.9% (n = 30), and 52.1% (n = 158) of patients received THA, 37.3% (n = 113) a bipolar HA and 10.6% (n = 32) received a cemented Thompsons prosthesis. Fixation of the femoral stem was cemented in 51.5% (n = 156) of patients (Table 2). The in-hospital mortality rate was 3.3% (n = 10) with a 2 year mortality rate of 35.3% (n = 107) (Table 3).

**Table 2.** Surgical Characteristics of Patients Undergoing Surgery.

Surgical variable	All patients N = 303% (n)
Surgical approach	
Posterior	61.4 (186)
Direct anterior	28.4 (86)
Antero-lateral	9.9 (30)
Type of surgery	
THA	52.1 (158)
Bipolar HA	37.3 (113)
Cemented thompson	10.6 (32)
Cemented vs uncemented (% cemented)	51.5 (156)

Data is presented as frequencies with counts in parentheses. THA, total hip arthroplasty; HA, hemi-arthroplasty.

**Table 3.** Frequencies of Combined Mortality Across Different Time Intervals Post-Surgery.

Mortality time-point	All patients N = 303% (n)
In-hospital	3.3 (10)
30 days post-surgery	5.6 (17)
90 days post-surgery	14.9 (45)
6 months post-surgery	21.4 (65)
12 months post-surgery	26.7 (81)
24 months post-surgery	35.3 (107)

Data is presented as frequencies with counts indicated in parentheses.

At 30 days post-surgery, an association between age and mortality was observed with patients who died being significantly older (81.6 ± 11.7 years) vs those that survived (73.3 ± 12.3 years) (OR 1.06, 95% CI: 1.02-1.12, *P* = .007) (Table 4). Similarly, undergoing a cemented procedure was predominant in the patients who died within 30 days following surgery, compared to those that did not (OR 3.02, 95% CI: 1.49-6.11, *P* = .002) (Table 4).

**Table 4.** Associations Between Risk Factors and Mortality at 30 Days Post-Surgery.

Risk factor	Died (n = 17)	Alive (n = 286)	Crude OR	*P*-value
	Percentage (n)	Percentage (n)	95% CI	
Age	81.6 ± 11.7	73.3 ± 12.3	1.06 (1.02-1.12)	.009
Gender				
Male	29.4 (5)	30.8 (88)		
Female	70.6 (12)	69.2 (198)	1.07 (.36-3.12)	.906
ASA score*				
1	.0 (0)	6.3 (18)		
2	17.6 (3)	48.3 (138)	.09 (.02-.51)	.006
3	64.7 (11)	40.0 (114)	.42 (.10-1.69)	.222
4	17.6 (3)	4.5 (13)		
Type of femoral fixation				
Uncemented	11.8 (2)	50.7 (145)		
Cemented	88.2 (15)	49.3 (141)	3.02 (1.49-6.11)	.002
Type of procedure				
Cemented thompson HA	29.4 (5)	9.4 (27)		
Cemented bipolar HA	52.9 (9)	32.2 (92)	.53 (.16-1.71)	.287
Hybrid THR	5.9 (1)	7.7 (22)	.25 (.03-2.26)	.215
Uncemented bipolar HA	0 (0)	4.2 (12)	––	
Uncemented THR	11.8 (2)	46.5 (133)	.08 (.01-.44)	.004
Surgical approach				
Posterior	58.8 (10)	61.8 (176)		
Antero-lateral	29.4 (5)	8.8 (25)	3.52 (1.11-11.14)	.032
Direct anterior	11.8 (2)	29.5 (84)	.42 (.09-1.96)	.268

Non-missing data is expressed as means ± standard deviations or frequencies with counts indicated in parentheses. ASA, American Society of Anaesthesiologist classification. *The ASA score of three participants was unknown.

At 6 months post-surgery, the association between age and mortality remained, with participants who died being significantly older (79.1 ± 11.8 years) vs those that did not (72.3 ± 12.2 years) (OR 1.05, 95% CI: 1.02-1.08, *P* < .001) (Table 5). Undergoing a cemented procedure was associated with 3.4 times increased odds of mortality against the uncemented procedure (OR3.40, 95% CI: 1.85-6.25, *P* < .001). Some independent associations were observed within the sub-categories of ASA score, type of procedure and the surgical approach, but none of these were included in the final multivariable model (Table 5).

**Table 5.** Associations Between Risk Factors and Mortality at 6 months Post-Surgery.

Risk factor	Died (n = 65)	Alive (n = 238)	Crude OR	*P*-value	AOR
	Percentage (n)	Percentage (n)	95% CI		95% CI
Age	79.1 ± 11.8	72.3 ± 12.2	1.05 (1.02-1.08)	<.001	1.03 (1.01-1.06)
Gender					
Male	27.7 (18)	31.5 (75)			
Female	72.3 (47)	68.5 (163)	1.20 (.65-2.21)	.554	
ASA score					
1	3.1 (2)	6.8 (16)			
2	28.1 (18)	52.1 (123)	1.17 (.25-5.52)	.842	
3	59.4 (38)	36.9 (87)	3.49 (.77-15.95)	.106	
4	9.4 (6)	4.2 (10)	4.80 (.81-28.60)	.085	
Type of femoral fixation					
Uncemented	26.2 (17)	54.6 (130)			
Cemented	73.9 (48)	45.4 (108)	3.40 (1.85-6.25)	<.001	2.48 (1.29-4.77)
Type of procedure					
Cemented thompson HA	20.0 (13)	8.0 (19)			
Cemented bipolar HA	47.7 (31)	29.4 (70)	.65 (.28-1.47)	.300	
Hybrid THR	6.2 (4)	8.0 (19)	.31 (.08-1.11)	.073	
Uncemented bipolar HA	4.6 (3)	3.8 (9)	.49 (.11-2.15)	.343	
Uncemented THR	21.5 (14)	50.8 (121)	.17 (.07-.41)	<.001	
Surgical approach					
Posterior	59.4 (38)	62.2 (148)			
Antero-lateral	17.2 (11)	8.0 (19)	2.25 (.99-5.14)	.053	
Direct anterior	23.4 (15)	29.8 (71)	.82 (.42-1.59)	.563	

Non-missing data is expressed as means ± standard deviations or frequencies with counts indicated in parentheses. ASA, American Society of Anaesthesiologist classification. *The ASA score of three participants was unknown.

An association between increased age and mortality was also observed at 12 months after surgery (OR 1.04 95% CI: 1.02-1.07, *P* < .001) (Table 5). Undergoing a cemented procedure was associated with a 2.95-times increased odds of mortality compared to being treated with an uncemented procedure (OR 2.5 95% CI: 1.71-5.09, *P* < .001). Again, various independent associations within the subcategories of the other predictor variables were observed during the univariable analysis, but none of these were included in the final multivariable model (Table 6) (*P* = .222).

**Table 6.** Associations Between Risk Factors and Mortality at 1 Year Post-Surgery.

Risk factor	Died (n = 81)	Alive (n = 222)	Crude OR	*P*-value	AOR
	Percentage (n)	Percentage (n)	95% CI		95% CI
Age	77.9 ± 11.9	72.2 ± 12.2	1.04 (1.02-1.07)	<.001	1.03 (1.00-1.05)
Gender					
Female	72.8 (59)	68.0 (151)			
Male	27.2 (22)	32.0 (71)	1.26 (.72-2.22)	.421	
ASA score					
1	2.5 (2)	7.3 (16)			
2	31.3 (25)	52.7 (116)	1.72 (.37-7.98)	.486	
3	57.5 (46)	35.9 (79)	4.66 (1.02-21.2)	.046	
4	8.8 (7)	4.1 (9)	6.22 (1.06-36.57)	.043	
Type of femoral fixation					
Cemented	70.4 (57)	45.6 (99)			
Uncemented	29.6 (24)	55.4 (123)	2.95 (1.71-5.09)	<.001	2.30 (1.28-4.13)
Type of procedure	16.0 (13)	8.6 (19)			
Cemented thompson HA	46.9 (38)	28.4 (63)			
Cemented bipolar HA	7.4 (6)	7.7 (17)	.88 (.39-1.98)	.761	
Hybrid THR	4.9 (4)	3.6 (8)	.52 (.16-1.66)	.267	
Uncemented bipolar HA	24.7 (20)	51.8 (115)	.73 (.18-2.94)	.659	
Uncemented THR			.25 (.11-.59)	.002	
Surgical approach					
Posterior	63.8 (51)	60.8 (135)			
Antero-lateral	13.8 (11)	8.6 (19)	1.53 (.68-3.44)	.301	
Direct anterior	22.5 (18)	30.6 (68)	.70 (.38-1.29)	.254	

Non-missing data is expressed as means ± standard deviations or frequencies with counts indicated in parentheses. ASA, American Society of Anaesthesiologist classification. *The ASA score of three participants was unknown.

The average length of stay (LOS) for the whole cohort was 12.3 ± 5.1 days (range 3.0-41.0 days) with patients going home having a LOS of 11.6 days vs patients needing stepdown 15.3 days and going to a care-facility 13.2 days. Most patients 73% (n = 221) were discharged to their pre-hospital dwelling, 15% (n = 45) needed interim step-down care and 10% (n = 29) were discharged back to their referral centre.

## Discussion

A recent systematic review by Downey et al reported the absence of published literature regarding hip fracture mortality in Africa.^7^ The results of this study demonstrate a 1 year mortality rate of 26.7%. This is higher than the current world average of 22.0% but equal to other studies that reported a 1 year mortality rate between 20 and 40%. However, our reported mortality rate is lower than the 29% from the UK published by Haleem et al.^9^ The 30 day mortality rate of our cohort (5.6%) is comparable to the published 30 day mortality rate in the UK of 5.7%.^19^

The lack of published mortality rates in Africa can partially be attributed to the absence of any national hip fracture database. Du Toit et al attempted to investigate mortality rates in patients who received hemiarthroplasty for neck of femur fractures and reported significantly higher mortality rates both at 30 days (12.5%) and 1 year (34.3%).^20^ However, this study is limited by an attrition rate of 38% and that patients who received THR were not included. The present study comprehensively evaluated all neck of femur fractures treated with arthroplasty and obtained mortality data directly from the South African Department of Home Affairs instead of relying on medical records.

Our institution is a tertiary academic hospital and the 2nd largest academic hospital in South Africa that provides dedicated FNF care for all the secondary and primary health facilities in a catchment area of 4 million people. The availability of tertiary anaesthetic service contributed to a low number of exclusions due to patients not being deemed fit-for-surgery.

To stratify risk in the present study, mortality was analysed between different risk factor groups during 3 time intervals: 30 days, 6 months and 1 year. This study did not have an a priori hypothesis of specific exposures resulting in mortality, but rather an observational approach to identify potential risk factors and generate hypotheses for future investigation.

Mortality evaluated at 30 days post-surgery highlighted that patient older than 80 years, and those that received a cemented femoral component had an increased risk of death. However, due the small sample size of patients who died at 30 days post-surgery these findings should be interpreted with caution. When mortality was however evaluated at 6 months post-surgery, patients age older than 79 years and undergoing a cemented femoral component still had an increased risk of death. Finally, when mortality was evaluated at 1-year post-surgery, the association with increased age and cemented procedures continued with the odds of death for those undergoing cemented procedures being nearly three times that of those undergoing uncemented procedures, when corrected for age 0.

The average age at presentation in this study (73 years) is younger than the average age of >80 years reported by authors from high-income countries.^5,6,8^ This finding is in agreement with the recent work of Dela et al who reported an average age of 75 years for South African patients.^14^ Neither Dela et al nor the present investigation could identify concrete evidence on why South African patients present at a lower age but can only speculate that this is mostly due to factors related to the quadruple burden of disease profile in South Africa. In the current study, men were observed to be significantly (*P* < .001) younger than women, however no statistical association between gender and mortality rates, when evaluated at different time intervals, were observed.

The relationship between ASA classification and increased risk of mortality is well described by previous authors.^6-8^ Although it would appear as though our patients followed the same trend of increased risk of mortality with an increase in ASA classification grading, ASA classification was not consistently observed to be associated with risk of mortality and was not included in our final multivariable models at 6- and 12-months. The Elixhauser index^29^ was used to categorize co morbidities of our patients but we did not include it in our multivariate analysis. Although the total sample size of our cohort is the largest reported to date in Africa, the total number of mortality events at each timepoint is low when one considers the multiple levels within the ASA category. Therefore, one needs to be mindful of the potential of a type II statistical error and interpret these results with caution.

Cemented Thompson prostheses had the highest frequency of mortality events at all time points. This is not a reflection of the type of prosthesis, but rather of the functional status of the patient, as cemented Thompsons was only selected for the non-walking patients. Various independent associations between type of procure and risk of mortality appeared to be present, but this variable was not included in the final multivariable logistic regression modelling process due to its inherent associations with other included variables including ASA score and the type of femoral fixation. It is tempting to suggest that cementing of the femur might potentially have a bigger influence than choice of implant (bipolar HA vs THR) on mortality rate.^21^ If one only considers cementation of the femur in isolation, it was independently associated with a significantly increased risk of mortality during all three time intervals (*P* = .002; *P* < .001; *P* < .001 at 30 days, 6 months and 1 year, respectively) and were included in both final multivariable models). Bone cement implantation syndrome (BCIS), although a highly controversial topic, could contribute to the early (in hospital and 30 days) mortality risk, BCIS is unlikely to contribute to increased mortality 6 months and 12 months. This finding may reflect the presence of a selection bias, as patients received cemented prostheses based on the quality of the femoral bone stock: patients with poor or deficient bone received a cemented prosthesis. The relationship between low bone mineral density at the hip joint and increased mortality has previously been described by Trived et al in elderly men.^22^ The findings of this study should therefore hopefully prompt further research.

The independent associations between surgical approach and mortality was an unexpected finding. Most of the cases were performed by the arthroplasty unit during daylight hours. This explains the predominance of the posterior approach (61.6%) as this is the preferred approach for elective hip surgery in our unit. The 86 cases that were performed via the DAA done during the end of the study period, were all part of the initial learning curve of the unit implementing the DAA for hip surgery. In this study cohort, patients receiving hip surgery via DAA appeared to have a lower mortality than those performed via the posterior or anterior-lateral approach but inequalities in sample sizes needs to be considered when interpreting this finding. Although not included in the final multivariable model. This absolute numbers that indicated a reduced mortality risk at 30 days and 6 month post-operative period, was not as apparent at 1 year post-surgery. We attribute this to the muscle sparing and minimally invasive surgical principles of the DAA,^26-28^ especially in the FNF patients that are at higher risk to be sarcopenic with poor muscle quantity and quality. The decreased post-operative pain and no functional limitations^28^ (sitting and bending of legs) incorporated into the rapid mobilization protocols implemented with DAA hip surgery, aided us to mobilize patients quicker and obtain an earlier discharge. The DAA has subsequently become the dedicated approach for FNF surgery in our hospital. This finding should however be replicated in the future and further investigated to determine whether there is a true association, or simply an observation due to chance.

The average LOS (12.3 days), as well as the proportion of patients who were discharged to their homes (73%), was shorter than the 34.6 days (15.1 days in acute followed by 19.5 days in trust hospital) with only 69% return to original residence, that was reported by in the National Hip Fracture Database (NHFD) annual report 2019.^19^ The average LOS requirement in Thailand, another upper middle-income country similar to South Africa, was reported to be 20.6 days whilst.^23^ In contrast, as published in the Australian and New Zealand hip fracture registry (ANZHFR) annual 2020 report, the average LOS in the acute surgical ward was much shorted with 6.4 days for New Zealand and 7.6 days for Australia, with only 15% and 13%, respectively, being discharged to their homes.^24^

We acknowledge potential limitations in our study such as the retrospective nature and the fact that it was conducted in only a single academic hospital. We further considered all-cause mortality data that has the potential to include deaths that are not related to hip fracture surgery. While the mortality data is very accurate, due to the retrospective review, the secondary findings must be reviewed with caution.

## Conclusion

This study reports the mortality rate post-surgery for hip fracture patients in our hospital to be comparable with international literature. Factors associated with a 1 year increased mortality was increasing age (age >79) and the use of a cemented prosthesis. Further research is needed to investigate other factors which may influence mortality rates in patients with femoral neck fractures.
